# Supplementary material for: Milk quality and milk transformation parameters from infected mammary glands depends on the infecting bacteria species
Source: PLoS One. 2019 Jul 1;14(7):e0213817. doi: 10.1371/journal.pone.0213817 (PMC6602173; doi:10.1371/journal.pone.0213817)
Supplement: S1 Fig — Curd weight (40% dry matter) at 24 h (g) in relation to curd firmness (CF; V) of cheese made at the laboratory, as described in detail by Katz et al. [15]. (DOCX) [file pone.0213817.s001.docx]

S1 Figure. Curd weight at 24 hours

Curd weight (40 % dry matter) at 24 h (g) in relation to curd firmness (CF; V) of cheese made at the laboratory, as described in detail by Katz et al. [15].
